# 

**Published:** 1903-07

**Authors:** 


### CONTENTS.

ORIGINAL COMMUNICATIONS—

On the Treatment of Internal Strabismus. By Dunbar Roy, M.D., Atlanta, Ga ..... 205
Our House and Our Servant. By H. McHatton, M.D., Macon, Ga ..... 212
X-Ray Treatment of Acne. By Henry R. Slack, Ph.M., M.D., LaGrange, Ga ..... 219
Abdominal Surgery in the Private Residence. By Marcus F. Carson, M.D., Griffin, Ga ..... 222
A Nephrectomy—With Remarks. By J. G. Earnest, M.D., Atlanta, Ga ..... 227
X-Rays in the Treatment of Malignant Growth. By Marcus F. Carson, M.D., Griffin, Ga ..... 229

EDITORIAL NOTES AND COMMENTS—

Dr. E. C. Thrash ..... 233
The Atlanta Mosquito Crusade ..... 234

NEWS AND NOTES ..... 235
CONTENTS CONTINUED ..... X

